# Magnetic Characterization of Direct-Write Free-Form Building Blocks for Artificial Magnetic 3D Lattices

**DOI:** 10.3390/ma11020289

**Published:** 2018-02-12

**Authors:** Mohanad K. I. Al Mamoori, Lukas Keller, Jonathan Pieper, Sven Barth, Robert Winkler, Harald Plank, Jens Müller, Michael Huth

**Affiliations:** 1 Institute of Physics, Goethe University, 60438 (M) Frankfurt, Germany; almamoori@physik.uni-frankfurt.de (M.K.I.A.M.); l.keller@physik.uni-frankfurt.de (L.K.); jpieper@stud.uni-frankfurt.de (J.P.); J.Mueller@Physik.uni-frankfurt.de (J.M.); 2 Institute of Materials Chemistry, Vienna University of Technology, 1060 Vienna, Austria; sven.barth@tuwien.ac.at; 3 Graz Centre for Electron Microscopy, 8010 Graz, Austria; robert.winkler@felmi-zfe.at; 4 Institute of Electron Microscopy and Nanoanalysis, Graz University of Technology, 8010 Graz, Austria; harald.plank@felmi-zfe.at

**Keywords:** focused electron beam induced deposition, micro-Hall magnetometry, magnetic nanostructures, micromagnetic simulations, artificial magnetic lattices

## Abstract

Three-dimensional (3D) nanomagnetism, where spin configurations extend into the vertical direction of a substrate plane allow for more complex, hierarchical systems and the design of novel magnetic effects. As an important step towards this goal, we have recently demonstrated the direct-write fabrication of freestanding ferromagnetic 3D nano-architectures of ferromagnetic CoFe in shapes of nano-tree and nano-cube structures by means of focused electron beam induced deposition. Here, we present a comprehensive characterization of the magnetic properties of these structures by local stray-field measurements using a high-resolution micro-Hall magnetometer. Measurements in a wide range of temperatures and different angles of the externally applied magnetic field with respect to the surface plane of the sensor are supported by corresponding micromagnetic simulations, which explain the overall switching behavior of in part rather complex magnetization configurations remarkably well. In particular, the simulations yield coercive and switching fields that are in good quantitative correspondence with the measured coercive and switching fields assuming a bulk metal content of 100 at % consisting of bcc Co${}_{3}$Fe. We show that thermally-unstable magnetization states can be repetitively prepared and their lifetime controlled at will, a prerequisite to realizing dynamic and thermally-active magnetic configurations if the building blocks are to be used in lattice structures.

## 1. Introduction

Three-dimensional (3D) nano-magnetism is an increasingly popular research topic that radiates into several fields of applied and fundamental research, as well as technological application [1,2,3,4]. At the same time, the fabrication and magnetic characterization of 3D nano-magnetic structures faces several methodical and technological challenges. With regard to fabrication several different routes have been developed, such as template-based methodologies [5,6,7,8], sophisticated electron beam lithography [9,10] and, recently, focused electron beam induced deposition (FEBID) as a direct-write technology for the realization of magnetic free-form structures as single elements or arrays [11,12,13,14,15,16,17]. Getting experimental access to the spatially resolved magnetic properties of 3D nanostructures is challenging for complex single-element nano-architectures and, in particular, for 3D arrays. Typically, a detailed understanding of the groundstate or metastable magnetization distributions in simple sample geometries is obtained from micromagnetic simulations [18]. Here, complex 3D nano-architectures can lead to demanding simulation scenarios. The magnetic stray-field coupling between different parts of 3D nano-architectures, in combination with geometrically imposed large curvatures in the magnetization distributions, require finely grained simulation meshes in parallel with large 3D simulation volumes [18].

In recent work, we have presented the FEBID nano-fabrication of magnetic 3D geometries in nano-tree or nano-cube shape, as well as their arrangement in small array structures [19]. By writing these structures, consisting of metallic Co${}_{3}$Fe, on custom-made micro-Hall sensor devices, we were able to measure their magnetic stray field component perpendicular to the sensor layer. We then employed an effective macro-spin model, complemented by micromagnetic simulations for selected configurations, to get some insight into the magnetic switching behavior of the nano-tree and nano-cube elements. We found in general that the macro-spin simulations provide a satisfying correspondence between the simulated switching fields and those observed in the micro-Hall experiments for several angles of the applied external field towards the sensor layer normal direction. However, the first micromagnetic simulation analysis presented in [19] already indicated that the magnetization reversal on the different edges of the nano-tree and nano-cube structures does not proceed via coherent rotation but via complex vortex-like magnetization configurations.

Here, we present a full account of the angle-dependent magnetic stray field measurements on the nano-cubes performed at several temperatures by means of micro-Hall magnetometry. We show how the magnetic configuration of single elements can be tuned towards a switching instability following a particular field-temperature sweep profile. We then determine the statistical survival probability of such a state, which is a pre-requisite for getting access to the magnetization dynamics in single elements and array structures by means of stray field measurements. A large part of our presentation is devoted to a detailed analysis of the magnetization distributions in close proximity to different switching instabilities based on micromagnetic simulations. In basic research, two important scientific questions to be addressed relate to the ground state of classical realizations of Heisenberg- and Ising-like spin Hamiltonians [20] and the physics of artificial spin ice [20,21,22,23,24,25,26,27]. We therefore discuss the implications of our findings with a view to using the nano-cube and nano-tree building block in artificial lattices.

## 2. Results

### 2.1. Geometry of Nano-Cubes and Nano-Trees

The focus of this paper is on the magnetic properties of nano-cube and nano-tree type magnetic building blocks. For this reason, we only briefly dwell on the geometric properties of the building blocks and refer to [19] with regard to more details concerning the microstructure and elemental composition analysis. The detailed study of the growth characteristics and shape evolution of 3D FEBID structures in general, also in dependence of the chosen precursor, is out of the scope of the present work. We refer to dedicated growth studies, such as [11,28,29], for more details. Nevertheless, we would like to briefly point out some aspects which are of specific relevance for magnetic deposits.

**Figure 1:**
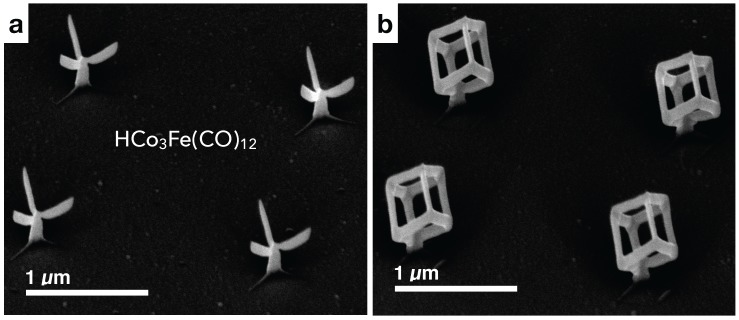
(**a**) SEM image of 2×2 array of non-coupled nano-trees. Note the varying cross section of the branches emanating from the vertical stem; (**b**) SEM image of 2×2 array of non-coupled nano-cubes.

In Figure 1, we show SEM images of the nano-tree and nano-cube type building blocks used in our magnetic study. We note two observations. First, depending on the beam energy, the cross sections of the various edges in the building blocks are elliptical with a degree of eccentricity e>0 that depends on the angle between the beam direction and the edge growth direction. For vertical pillars e=0 by symmetry. *e* grows as the tilt angle of the edge gets larger and is getting larger the larger the beam energy. This is due to the fact that for larger beam energies the primary electrons’ penetration depth exceeds the edge diameter (see [29] for details). Second, for the magnetic 3D structures, employing the precursor HCo${}_{3}$Fe(CO)${}_{12}$, we commonly observe a reduced cross section of tilted edges that connect to a vertical pillar. This reduction is not symmetric but most pronounced at the lowest position of the junction between pillar and tilted edge (see, in particular, Figure 1a). As the growth of the tilted edge proceeds, the cross section increases, becomes stationary and eventually tapers off as the end of the edge is reached. Presently, we assume that the magnetic stray field emanating from the pillar causes a slight deflection of the electron beam. This interaction gets smaller as the length of the edge is increasing during growth. Since the deposition process is done in the immersion mode of the objective lens, a magnetic field of about 70 mT oriented along the pillar direction is always present. We have verified this by independent Hall sensor measurements in our setup. It is therefore to be expected that the pillar is magnetically in a fully saturated state, which will cause the strongest possible stray field in beam direction resulting in a sideways deflection of the electron beam. Careful visual inspection reveals that this effect, but much less pronounced, is also observed for the nano-cubes where the tilted edges are connected to the vertical pillar.

For the micro-magnetic simulations presented below, we take these geometrical subtleties not into account but rely on a simplified geometry in the spirit of an effective model. In addition, we focus on the magnetic properties of the nano-cubes and refer to [19] for more details concerning the nano-trees.

### 2.2. Temperature-Dependent Magnetization Switching

**Figure 2:**
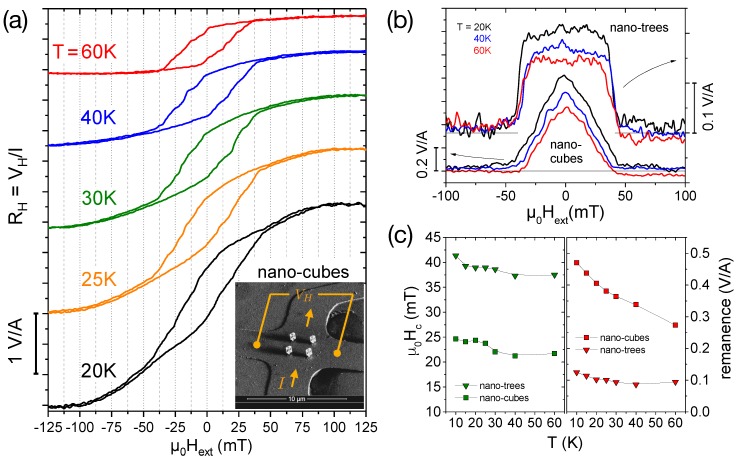
(**a**) Temperature dependence of the CoFe nanocubes’ magnetic hysteresis loop. Inset shows an SEM micrograph of the corresponding Hall cross and the Hall measurement configuration; (**b**) ’Area’ of the hysteresis loop (up- minus down-sweep) for the nano-cubes and -trees at selected temperatures; (**c**) Temperature dependence of the switching field (**left**) and zero-field stray field magnitude (**right**) corresponding to coercivity and remanence, respectively, of both nano-cubes and -trees.

Figure 2a shows the CoFe nano-cubes’ magnetic hysteresis loop as Hall resistance $R_{H}\equiv V_{H}/I\propto \langle B_{z}\rangle$ vs. $\mu_{0}H_{\mathrm{ext}}$ at different temperatures for the external magnetic field perpendicular to the sensor plane ($\theta =0^{\circ }$), i.e., parallel to the stem of the structures. Clear steps in the stray-field response to changes of the external field are observed. As discussed in [19], a simple macro-spin approach—where stem and edges of the nano-cubes are represented by a single, macroscopic spin moment with uniaxial anisotropy based on the assumption that all microscopic magnetic moments within the macrospin point in the same direction and rotate collectively—reveals that an almost vertical decrease or increase of $\langle B_{z}\rangle$ of the simulated up- and down-sweep curves (see Figure 1b in [19]), respectively, is associated with the rotation of the four stems, whereas smaller steps are caused by the almost simultaneous flipping and canting of the edge macro-spins. In comparison to these macro-spin simulations, the more rounded and sheared hysteresis loops with smaller area and coercive field observed in the experiments shown in Figure 2a point to a non-uniform magnetization reversal of the stems dominated by multi-domain switching events, which is confirmed by micromagnetic simulations (see Section 2.3 below for a detailed discussion). Furthermore, it is noteworthy that for lower temperatures the hysteresis loop exhibits a significant S-shaped rounding accompanied by a slight opening of the hysteresis loop, which—after reducing the external field from saturation—sets in at higher fields for lower temperatures. This effect is most pronounced at low temperatures, e.g., T=20 and 25 K, and has almost vanished at the highest temperature of our measurements of 60 K.

In order to quantify the temperature dependence of the switching field (coercivity) and the magnitude of the measured stray field, we analyze the ’area’ of the hysteresis loop calculated by subtracting the up- from the down-sweep curves. Note that any residual nonlinear background contribution (caused by the fact that the cross with the magnetic structure and the empty reference cross (see a description of our gradiometry setup in Section 4.2) are not perfectly identical) cancels out in this procedure. Figure 2b compares these ’area’-curves at three selected temperatures for the nano-cubes and nano-trees (hysteresis loops for the latter not shown here (see [19] for the curves at T=30 K)). For the cube structures, the ’areas’ have a triangular, peak-like shape, whereas, for the trees, the hysteresis loops are more square-shaped, related to the fact that stem and branches have predominantly easy-axis magnetization components parallel to the externally applied field. The flux coupling through the three-leg vertices at the corners connecting the edges of the nano-cubes leads to step-wise switching behavior, whereas for the nano-trees a gradual rotational change of the magnetization direction is followed by a sharp switching process comprising a substantial part of the sample volume [19].

Figure 2c shows the temperature dependence of the coercive field and the remanence determined from the ’area’ curves as shown in Figure 2b by taking half the value of the full-width-at-half-maximum and the zero-field value, respectively. The coercive fields—with the square-shaped hysteresis loops of the nano-trees naturally yielding larger values—show a small but significant decrease with increasing temperature manifested by a rounded step to smaller values at around 30 K, which is most pronounced for the nano-cubes, and apparently related to a change in the magnetic anisotropy. At the same temperature, there is also a change of slope in the remanence vs. temperature. Although this behavior is currently not understood, it is interesting to note that a similar step in the switching field vs. temperature at about the same temperatures has been observed for arrays of nanometer-sized Fe particles [30] grown by scanning tunneling microscopy assisted deposition, a related fabrication technique, for diameters of the cylinder-shaped particles larger than the critical value for a single-domain state.

The remanent stray field—due to the magnetic volume being larger for the cubes—decreases with increasing temperature for both nano-trees and -cubes. For the former, however, this effect is relatively small, whereas for the latter the remanence at T=60 K is reduced to about half the (extrapolated) value at T=0 K. This may be qualitatively understood by considering the differences in the number of energetically different magnetization states of the nano-trees and -cubes if one considers just simple uniform magnetization along the edges. Whereas for the trees only three discrete energy levels exist, a much larger number of energetically equivalent magnetization states occur for the nano-cubes with stem. These configurations lie relatively close in energy and excited (demagnetized) states with smaller total *z*-components of the magnetic stray field are easily thermally populated.

**Figure 3:**
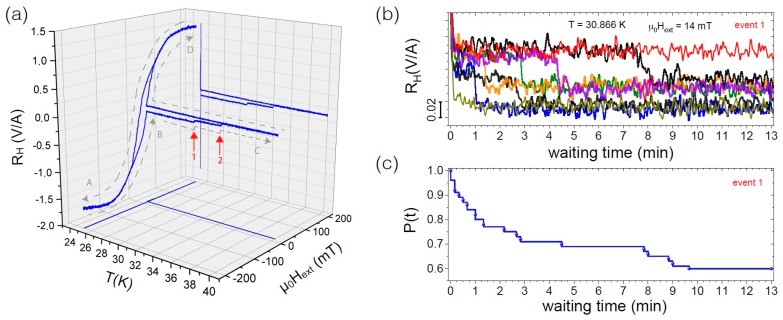
(**a**) Temperature and magnetic field protocol for thermal activation of a single switching event within the nano-cubes’ hysteresis loop taken at $\theta =0^{\circ }$. A: Saturation at negative fields at T=25 K. B: Preparation of the state at $\mu_{0}H_{\mathrm{ext}}=14$ mT. C: Two major thermally activated switching events are observed (red arrows) while warming up to T=40 K in a fixed field of 14 mT. D: Cool-down and read out of the accessed state by completing the hysteresis loop at T=25 K; (**b**) Time dependence of a repeatedly prepared state being thermally unstable at a temperature of 30.866 K (selected curves for switching event 1); (**c**) Occupation number P(t) of the so-prepared state. The dots mark the time after which each single switching event of a total of 50 consecutively identically prepared states occurs. Only events that were observed within 13 min are recorded.

In view of further developing the present structures towards a more Ising-like switching behavior (see discussion below), which may allow for thermally active networks of 3D magnetic nanostructures, it is instructive to further examine the temperature dependence of the nano-cubes’ switching dynamics by preparing a state inside the hysteresis loop measured at T=25 K in close vicinity of an expected switching event at $\mu_{0}H_{\mathrm{ext}}=14$ mT after saturation at negative fields. While keeping $H_{\mathrm{ext}}$ constant, the sample temperature was increased from T=25 K up to 40 K while monitoring its stray field. As shown in Figure 3a, we observe two distinct steps while warming up, corresponding to subsequent states in the magnetic hysteresis loop. Upon subsequent cool down from 40 K, the system stays in the so-prepared state, which is read out by completing the hysteresis loop. The system was then prepared multiple times in a state at T=30.866 K and $\mu_{0}H_{\mathrm{ext}}=14$ mT, where a spontaneous transition (state 1, indicated by red arrow) is expected to occur within an experimentally accessible timescale of a few minutes. In 20 out of 50 such time-dependent measurements, the transition into the energetically more favorable state was observed within 13 min (see Figure 3b). Thin curves represent runs, where no switching event occurred. We have performed a similar study on other FEBID-fabricated samples, namely a single 2D Co nanoelement and a 2D nanocluster building block of artificial square spin ice, i.e., an ensemble of twelve Co nanoelements [31,32], where we systematically investigated the decay of a multiple-prepared state as a function of temperature and magnetic field. In [31] we demonstrate that even a single, in the static sense mono-domain, nano-element already exhibits a complex switching behavior. For a cluster of twelve such mono-domain elements, we found that different configuration paths play a role for the switching dynamics, i.e., stable microstate configurations, which are statistically accessed during magnetic reversal [32]. In Figure 3c, for our 3D CoFe nano-cubes, we exemplarily show the ’survival function’ P(t) of the state prepared as described above. P(t) corresponds to the occupation number of the state, i.e., the system’s probability of not having switched after a time *t*. At each switching time, P(t) is reduced by 1/50 corresponding to a reduction of the probability of not having switched at this time. Although more than one of the edge elements, possibly including a magnetic vortex state, might be involved in the switching and assuming that the switching occurs from slightly different initial configurations/microstates of the remaining elements and to different final microstates, based on the observation that the measured reduction of the stray field is the same, it is reasonable to assume that the transition can be ascribed to a single switching process. We note that the observed P(t) for the selected temperature and magnetic field values cannot be described by a single exponential or stretched survival function based on a simple Néel-Brown model as seen in ideal single-energy barrier systems under thermal perturbation [33] or in more complex configuration landscapes, respectively. We rather observe an initial strong decay of the magnetization state at short times, which seemingly saturates before—at longer times—the occupation number shows a stronger decrease again, indicating an additional decay channel. A disagreement with the Néel-Brown model is expected considering the metal-core/oxide-shell structure of our 3D nano-cubes [33]. In addition, the rather complex switching behavior, which is confirmed by micromagnetic simulations (see below), but also frustrated interaction effects may affect the magnetization reversal of the nano-cube clusters.

### 2.3. Angular-Dependent Magnetization Switching—Experiment and Micromagnetic Simulation

**Figure 4:**
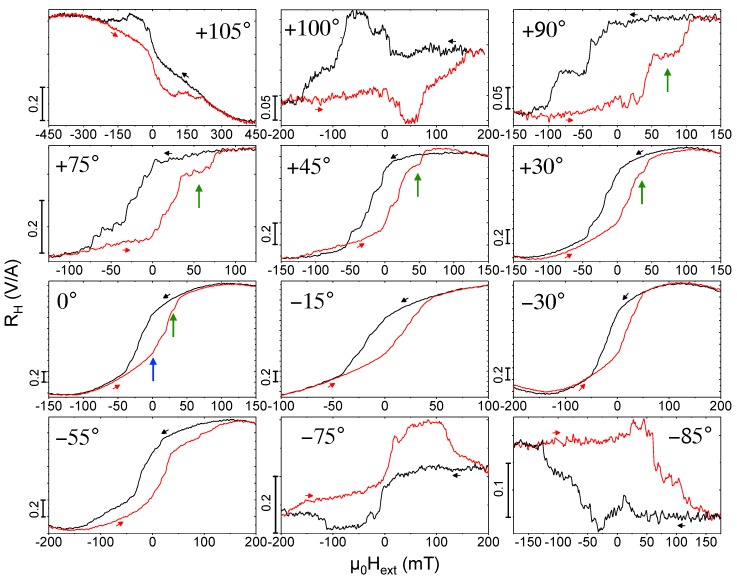
Angular dependence of the magnetic hysteresis loops at T=30 K shown as $R_{H}\equiv V_{H}/I$ vs. $\mu_{0}H_{\mathrm{ext}}$ for CoFe nano-cubes, obtained for the indicated values of the angle θ of the external applied magnetic field relative to the surface normal of the Hall sensor. Note the varying scales of stray field and external field axes. Black and red arrows indicate down- and up-sweep curves, respectively, following initial saturation at positive field values. Blue and green arrows indicate characteristic switching events and magnetization configurations described in the text.

**Figure 5:**
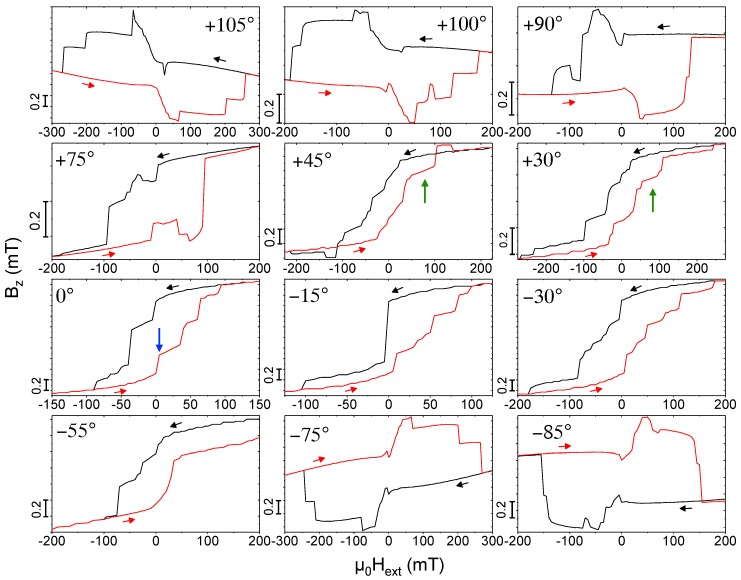
Angular dependence of the magnetic hysteresis loops shown as the magnetic stray field $\langle B_{z}\rangle$ *vs.* $\mu_{0}H_{\mathrm{ext}}$ calculated from micromagnetic simulations for the same angles as in Figure 4. Blue and green arrows indicate characteristic switching events and magnetization configurations described in the text.

In simple magnetic structures, as e.g., nanowires with a single anisotropy axis, the angular dependence of the switching fields reveals important information on the mechanism of magnetization reversal [34,35]. In the following, we discuss the angular dependence of the magnetization reversal of the present 3D CoFe nano-structures and compare the experimental hysteresis loops in Figure 4 with micromagnetic simulations in Figure 5 exemplarily for the nano-cubes. For details regarding measured and simulated hysteresis curves for the nano-trees at selected angles, see [19]. In [19] we also have presented a detailed microstructural characterization of the structures yielding a metallic Co${}_{3}$Fe core that is surrounded by a metal-oxide sheath that is a ferrimagnetic spinel phase. For the micromagnetic simulations discussed here, we consider full-metal deposits of Co${}_{3}$Fe but have taken diameter values that are reduced as compared to the geometrical diameter of the structures as revealed from SEM and TEM imaging. We find favorable correspondence between the angular-dependent characteristic switching fields and remanent stray fields, as well as the shapes of hysteresis loops between simulation results and micro-Hall magnetometry data (see also Section 3 below).

Figure 4 shows the measured Hall resistance, representing the magnetic stray field $\langle B_{z}\rangle$, as a function of the external magnetic field $H_{\mathrm{ext}}$ applied at different angles with respect to the sensor plane, with $\theta =0^{\circ }$ corresponding to $H_{\mathrm{ext}}$ perpendicular to the 2DEG plane and parallel to the nano-cubes’ stem. (The curves taken at $\theta =0^{\circ }$, +45${}^{\circ }$ and +105${}^{\circ }$ are the same data as in [19].) We observe a systematic change of the characteristic step-like switching behavior with varying field angles. The micromagnetic simulations help with identifying the relevant processes and assigning characteristic features by evaluating the microscopic magnetization distribution for different points within the hysteresis loops. For $\theta =0^{\circ }$, at $H_{\mathrm{ext}}=-150$ mT, i.e., in the reversible, nearly saturated state, the *z*-axis magnetization $m_{z}$ almost uniformly points downwards along the anisotropy axes of the nano-cube’s stem and edges. Upon increasing the external field, a magnetic vortex state starts to form predominantly along the stem and vortices nucleate at the three-leg vertices of the edges with curled magnetization along the edges giving rise to the opening of the hysteresis. The demagnetized state of the up-sweep curve then shows curled (vortex) structures essentially forming in all edges and the stem with the overall *z*-component of the magnetization still pointing downwards. Upon further increasing the external field to positive values, a sudden, large upward jump is seen in the simulations corresponding to a pronounced kink in the measured stray field close to $H_{\mathrm{ext}}=0$ (see blue arrows in Figure 4 and Figure 5 ($\theta =0^{\circ }$), which is caused by the simultaneous switching of $m_{z}$ of three edges of the cube behaving like a bistable nano-rod and a substantial curling of the stem’s magnetization and that of the three other edges. Upon further increasing field, the following large jump in the simulated hysteresis loop is caused by the switching of three more edges and the third one leads to a state where $m_{z}$ of all edges except two have been switched. Finally, accompanied by the formation of magnetic vortex configurations going through the edges, $m_{z}$ of the two remaining edges switches and at positive ’saturation’ of +150 mT the magnetization direction of the stem and all edges point along their anisotropy axes in the positive *z*-direction. Naturally, the experimentally observed hysteresis loop is more rounded, but the essential switching processes can be identified.

From this multi-step switching at zero field angle, the experiment shows for increasing positive angle the formation of a pronounced plateau before the final large positive switching event occurs in the up-sweep curves. This plateau is highlighted by green arrows in Figure 4 and Figure 5 for θ = +30${}^{\circ }$, $+45^{\circ }$, +75${}^{\circ }$, +90${}^{\circ }$. Remarkably, the simulations reproduce this and related features like the wide opening of the hysteresis loop for θ = +30${}^{\circ }$ (also observed for $\theta =-55^{\circ }$) and the crossing of the up- and down-sweep curves for an angle of +45${}^{\circ }$ very well. The origin of the irreversible magnetization reversal at rather large external fields for the angle of θ = +30${}^{\circ }$ is a large vortex structure forming along the long axes of the nano-cube’s three upper edges (not connected to the stem), the anisotropy axes of which are pointing towards the direction of the external field at a small angle. In the up-sweep curve, these vortex structures retain a stronger curling with a larger negative $m_{z}$-component compared to the down-sweep at the same fields. The angle between $H_{\mathrm{ext}}$ and the anisotropy axis of four of the edges then becomes almost zero for an angle of +45${}^{\circ }$, where the crossing of the up- and down-sweep curves very likely is again caused by the difference in the magnetic vortex confuguration in one of the edges parallel to the applied field.

**Figure 6:**
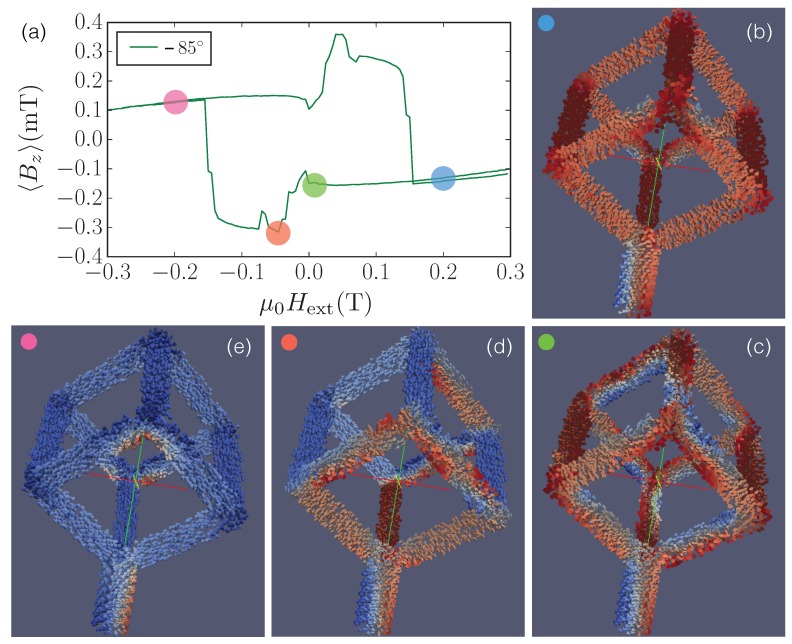
(**a**) Simulated hysteresis loop for $\theta =-85^{\circ }$; (**b**–**e**) Magnetization distribution represented by the *y*-component of the magnetization (red color: +1, blue: −1) at different positions in the hysteresis loop indicated by colored discs.

For large angles, beginning at about $\pm 75^{\circ }$, the simulated hysteresis curves develop a pinching effect with an up- and downward switching and a corresponding broad maximum (down sweep) and minimum (up sweep) of the stray field/magnetization in the negative and positive field ranges, respectively. As an example, we discuss the switching behavior and show the micromagnetic configurations for an angle $\theta =-85^{\circ }$, where the simulations shown in Figure 6a qualitatively reproduce the measured hysteresis loop shown in Figure 4 quite well. Figure 6b–e display a 3D view of the magnetization direction at selected external magnetic fields. Color-coded (red: +1, blue: −1) is the *y*-component of the magnetization $m_{y}$, where the arrowheads represent the magnetization direction (every 20th simulation cell is shown). The external magnetic field lies in the *y*–*z*-plane (yellow and green coordinates). Several characteristic features are noteworthy. First, at all external fields shown here, see, for example, $\mu_{0}H_{\mathrm{ext}}=\pm 0.2$ T, where the magnetization has fully switched, the nano-cubes’ magnetization is not saturated but the stem retains a vortex state as well as the three-leg vertices. Second, the switching of individual edges are clearly visible (see, e.g., the changes in magnetization configurations when $\mu_{0}H_{\mathrm{ext}}=0\;T\to -0.05\;T\to -0.2\;T$). Third, as discussed for other angles above, the switching of the edges’ magnetization develops from a vortex-like magnetization distribution.

## 3. Discussion

**Figure 7:**
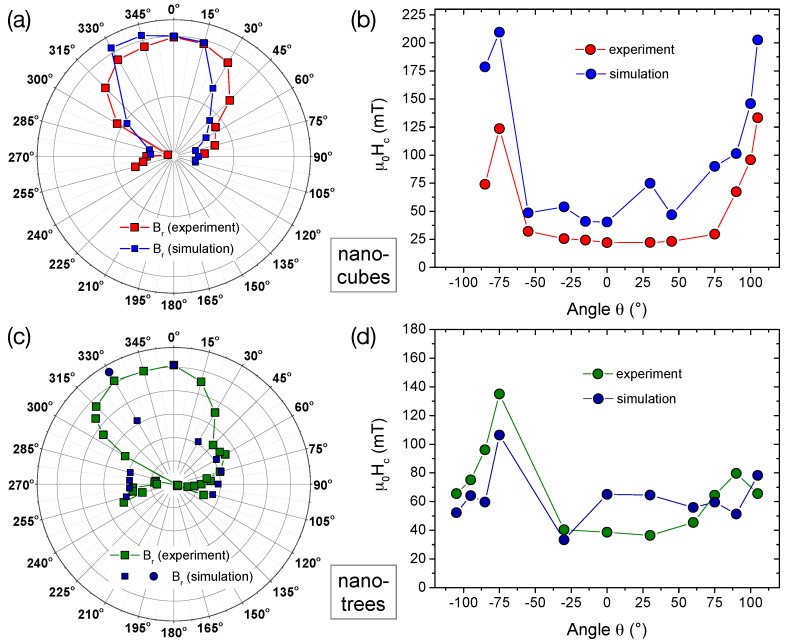
(**a**,**c**) polar plots of the angular dependences of the remanent magnetization (stray field $B_{r}$) comparing the experimental and simulated results (scaled to the values at $\theta =0^{\circ }$) for the nano-cubes and nano-trees, respectively; (**b**,**d**) linear plots of the angular dependences of the measured and simulated ’coercive fields’ $\mu_{0}H_{c}$ determined by evaluating the ’area’ curves (down-sweep minus up-sweep) and taking half the value of the full-width-at-half-maximum for both nano-cubes and -trees. Lines are guides to the eyes. (At $\theta =-30^{\circ }$, this method yields a too small value for the simulated hysteresis, likely due to the configuration being ’trapped’ in a metastable state. Reading just the value in the demagnetized state for the down-sweep curves, circle in (**c**), results in a better correspondence with the experiment.)

The results comparing the magnetization reversal for different field angles between the micro-Hall measurements and the micromagnetic simulations are summarized in Figure 7 for both the CoFe nano-cubes and nano-trees.

Figure 7a shows the angular dependences of measured and simulated remanent stray fields $B_{r}$ in a polar plot, scaled to the values at $\theta =0^{\circ }$, and Figure 7b the comparison of the coercive fields $\mu_{0}H_{c}$ for the CoFe nano-cubes. As described above, for the latter, the full-width-at-half-maximum of the hysteresis’ area has been used. For both quantities, the qualitative agreement between experiment and simulation is remarkably good. For the remanence $B_{r}\left(\theta \right),$ the measured two-fold symmetry is well reproduced by the simulations, although the angular distribution seems slightly narrower. In addition, the relative changes in the coercivity $\mu_{0}H_{c}$ with angle do agree quite well, although the absolute values are somewhat larger in the simulations. The switching fields at lower angles are much smaller than for larger angles. This can be explained by the dominating contribution of the nano-cubes stem with an inhomogeneous magnetization distribution involving creation and annihilation of magnetic vortices as described above.

For the nano-trees, the angular dependence and even the measured absolute values of $\mu_{0}H_{c}\left(\theta \right)$ correspond well with the simulations assuming a bulk metal content of 100 at% consisting of Co${}_{3}$Fe (bcc), thus confirming that the metal content is not significantly reduced below at least the 80 to 90 at% level.

An interesting application of the building blocks presented here is their use in artificial lattice structures. In [19] we have demonstrated that diamond-like artificial lattices can be prepared by FEBID using the nano-tree as building block. Such lattices can be expected to show highly complex magnetization distributions. Our magnetic analysis reveals rather complex spatial magnetization distributions in the individual building blocks depending on the direction and magnitude of the applied magnetic field. It might be possible to suppress the occurrence of curling magnetization states in zero field if the junctions between the edges of the building blocks can be made from non-magnetic material, as has been demonstrated to be possible in [19]. In addition, the diameter of the roughly cylindrical magnetic edges will have to remain below about five to seven times the exchange length, i.e., about 20 to 30 nm in the present case, to ensure a ground state with uniform magnetization [36]. This has to remain as a true challenge for 3D FEBID of magnetic nano-structures to be addressed in future work.

## 4. Materials and Methods

### 4.1. Focused Electron Beam Induced Deposition

Samples were fabricated using a dual beam SEM/FIB (FEI, Nova NanoLab 600, Frankfurt, Germany), equipped with a Schottky electron emitter operating at a base pressure of about $2\times 10^{-7}\;$mbar. The precursor HCo${}_{3}$Fe(CO)${}_{12}$ was injected in the SEM by means of a capillary with an inner diameter of 0.5 mm. The distance capillary-surface was about 100μ m and the tilting angle of the injector was $50^{\circ }$. The crucible temperature of the gas injection system (GIS) was set to 65 °C. The electron beam parameters used during deposition were 20 keV for the acceleration voltage and 13 pA for the beam current. The dwell time was set after optimization of the 3D growth to 1ms. The pitches depend on the inclination angle of the 3D structures and have to be adapted to the precursor and gas flow conditions [19]. Concerning the synthesis of the Co-Fe precursor, we refer to [37]. As detailed in [19], we found the co-deposit or halo to have no significant influence on the switching behavior of the magnetic 3D structures.

### 4.2. Micro-Hall Magnetometry

The magnetic stray field of a sample is directly linked to its magnetization [38] and is measured by detecting the Hall voltage $V_{H}$ in the sensor plane formed by a two-dimensional electron gas (2DEG) at the interface of a AlGaAs/GaAs heterostructure (see schematics in Figure 2a). An array of six 5×5 μm${}^{2}$ Hall crosses is patterned by standard UV lithography followed by wet chemical etching. The 2DEG is electrically contacted by annealed AuGe/Ni contact pads and gold wire bonding. A Cr/Au top-gate allows for adjusting the sensor’s carrier concentration *n* and served as the substrate for 2×2 arrays of CoFe nano-cubes and -trees written by FEBID. After writing the 3D magnetic nanostructures directly onto the Hall crosses, the sensor has been transferred in less than one hour to a cryogenic system in a low-pressure He atmosphere. The sample can be rotated with respect to the external magnetic field, $\mu_{0}H_{ext}$, of a superconducting magnet essentially free of magnetic flux jumps, where $\theta =0^{\circ }$ corresponds to the field applied perpendicular to the sensor plane.

In first approximation, cf. Equations (1)–(3), the detected *z*-component of the stray field averaged over the active area of the Hall-cross, $\langle B_{z}\rangle$, is directly proportional to the measured Hall voltage $V_{H}$. Since the integrated stray field of the arrays of nano-cubes or -trees grown on top of the Hall sensor is more than two orders of magnitude smaller than the applied external field $\mu_{0}H_{ext}$, a so-called gradiometry measurement is performed, where the large Hall-effect background signal being linear in $\mu_{0}H_{ext}$, is cancelled in situ by the differential measurement of an empty Hall cross. This results in a stray field contribution of the magnetic nanostructures given by

(1) $$\Delta V_{H}=\frac{1}{ne}\cdot I\cdot \langle B_{z}\rangle \;,$$

with $n=3.4\times 10^{11}\;{\mathrm{cm}}^{-2}$ and an applied current of I=2.5μA. We note that the temperature dependence of the carrier concentration is very weak for temperatures below about 100 K [39,40]. The stray field $\langle B_{z}\rangle$ is detected in the active area *A* of the Hall cross in the plane of the 2DEG buried about 115 nm below the Cr/Au top-gate. Data have been corrected by subtracting a small linear background caused by slight differences between the two crosses in the gradiometry setup.

In this work, we show the Hall magnetometry data as $R_{H}\equiv V_{H}/I$ vs. $\mu_{0}H_{ext}$ implying that the simple Equation (1) may fail to describe (i) a Hall response function $F_{H}\left(x,y\right)\neq 1$ in the diffusive transport regime [41], where

(2) $$V_{H}=\frac{1}{ne}\cdot I\cdot \frac{\int_{A}dxdyB_{z}\left(x,y\right)F_{H}\left(x,y\right)}{\int_{A}dxdyF_{H}\left(x,y\right)},$$

and (ii) the influence of the inhomogeneous magnetic field distribution and the mean free path of the electrons in the 2DEG in the ballistic regime resulting in a correction factor α [42]:

(3) $$V_{H}=\alpha \cdot \frac{1}{ne}\cdot I\cdot \langle B_{z}\rangle .$$

With the carrier concentration and mobility at T=30 K in the present experiment of $n=3.4\times 10^{11}$ cm${}^{-2}$ and $\mu =4.5\times 10^{5}$ cm${}^{2}$/Vs, respectively, an effective mean free path of the electrons of $l_{\mathrm{eff}}∼4.3\;\mu m$ can be estimated. This is still smaller but close to the structures’ size of nominally w=5μm (neglecting any edge depletion); therefore, the transport may be at the crossover of quasi-ballistic to diffusive regime. For the latter, the correction factor, which reflects the response function $F_{H}\left(x,y\right)$, usually underestimates $\langle B_{z}\rangle$, i.e., α<1. For the intermediate transport regime, a rigorous theoretical description for the correction factor as a function of $w/l_{\mathrm{eff}}$ does not exist, but values between 0.5 and 1 are expected. However, it has been shown that α depends on the field strength, the level of magnetic field non-uniformity and the shape of the Hall cross. For example, the increase of the Hall crosses’ active area due to circular corners must be considered, which leads to a further reduction of the measured Hall signal.

In comparison to the micromagnetic simulations of the stray fields calculated in this work, assuming a bulk metal content of 100 at% consisting of Co${}_{3}$Fe (bcc), we find a correction factor of α≈0.45 for the external field perpendicular to the sensor, when extrapolating the remanent stray field to T=0 K (see Figure 2d). We note that such a value can already be accounted for by merely taking the upper limit of the expected signal reduction due to the rounded corners of the Hall crosses.

### 4.3. Micromagnetic Simulations

Micromagnetic simulations at T=0 were performed by numerically solving the Landau-Lifshitz equation

(4) $$\frac{\partial {\vec{S}}_{i}}{\partial t}=-\gamma \frac{\partial H}{\partial {\vec{S}}_{i}}\times {\vec{S}}_{i}-\alpha \gamma \left(\frac{\partial H}{\partial {\vec{S}}_{i}}\times {\vec{S}}_{i}\right)\times {\vec{S}}_{i}\;$$

for a single nano-tree/cube consisting of Co${}_{3}$Fe. Equation (4) describes the motion of a spin ${\vec{S}}_{i}$ of unit length at site *i* caused by an effective field ${\vec{H}}_{\mathrm{eff}}$ that is generated by the interactions modeled by a Heisenberg Hamiltonian complemented with magnetic dipolar interactions and coupling to an external field

(5) $$H=-D\sum_{i=1}{\left(\mu_{i}{\vec{S}}_{i}\cdot {\vec{e}}_{i}\right)}^{2}-\frac{\mu_{0}}{4\pi }\sum_{i<j}\mu_{i}\mu_{j}\frac{3\left({\vec{S}}_{i}\cdot {\vec{e}}_{i,j}\right)\left({\vec{e}}_{i,j}\cdot {\vec{S}}_{j}\right)-{\vec{S}}_{i}\cdot {\vec{S}}_{j}}{r_{i,j}^{3}}-{\vec{B}}_{\mathrm{ext}}\cdot \sum_{i=1}\mu_{i}{\vec{S}}_{i}\;.$$

In Equation (4), γ denotes the gyromagnetic ratio and α is a phenomenological damping factor.

From a careful EELS analysis presented in our previous work [19], we found clear evidence for the formation of Co-Fe-oxide on the surface of the nano-cubes, whereas the bulk of the structures consists of essentially pure metallic Co${}_{3}$Fe. By comparing micromagnetic simulations assuming a metal-core/oxide-shell structure with simulations of purely metallic Co${}_{3}$Fe, we found that the oxide-shell leads to small changes of the switching fields, which is caused by the much smaller saturation magnetization of the oxide as compared to bulk Co${}_{3}$Fe (see [19] for details). Here, we use for our micromagnetic simulations an effective material model without core-shell structure but assume reduced diameters, as compared to the geometrical diameters deduced from SEM and TEM inspection, for the stem and edge of a fully metallic Co${}_{3}$Fe nano-tree/cube.

In our simulations, we use cubic voxels of edge length 5 nm for the finite difference discretization in MuMax3, a GPU-accelerated micromagnetic simulation program [43]. We have verified by comparison to high-resolution simulations with 2.5 nm edge length (about half of the exchange length) for selected angles that the results obtained for the the field-dependent magnetization and stray fields are almost identical. The other simulation parameters were chosen as follows:

**Nano-tree**  *Geometrical dimensions*: stem diameter $D_{s}=100$ nm (cylindrical) and length $L_{s}=185$ nm, edge diameters $D_{e,1}=60$ nm and $D_{e,2}=48$ nm (elliptical) at a length of $L_{e}=340$ nm. The longer semi-axis with diameter $D_{e,1}$ is roughly in the beam direction (see [19] for details). *Material parameters*: saturation magnetization $M_{S}=1.5\times 10^{6}\;$A/m and exchange constant $A=1.5\times 10^{-11}\;$J/m by averaging the respective value for Fe and Co [36,38].
**Nano-cube**  *Geometrical dimensions*: stem diameter $D_{s}=100$ nm (cylindrical) and length $L_{s}=185$ nm, edge diameter $D_{e}=60$ nm (cylindrical) at a length of $L_{b}=340$ nm. *Material parameters*: see material parameters for nano-tree.

The nano-crystalline/granular microstructure of the deposits leads to an averaging of the magnetic anisotropy, which is why we have omitted any anisotropy energy contributions in our simulations. In order to guarantee sufficiently fast convergence, we set the damping parameter α to 0.3, use the full relaxation of MuMax3 [43] at the initial field value and then the conjugated gradient method for quicker convergence at all other field values of each cycle with a stop criterion of $10^{-6}$.

The simulation data on the orientation of the magnetic moments within the voxel elements of the nano-tree/cube for each external field was used to calculate the average stray field $\langle B_{z}\rangle$ at the position of the sensor layer as follows:

1. The positions of the four nano-trees and nano-cubes on the Hall sensor area were determined from SEM images.
2. For each voxel element of the nano-tree/cube, the associated simulated magnetic moment was used to calculate the corresponding dipolar stray field. The stray field contributions of all moments of the nano-tree/cube set to one of the four positions were averaged over n×n positions of the sensor array area (roughly 5×5μm${}^{2}$) in the xy-plane at the *z*-position of the 2DEG sensor 115 nm below the substrate surface.
3. The resulting four averaged stray fields were added to obtain the full averaged stray field of the four nano-trees/cubes.

We checked that this coarse-grain averaging to obtain the overall stray field acting at the 2DEG position showed no appreciable changes anymore for n≥28, which is why we set n=28.

## 5. Conclusions

3D fabrication of magnetic nano-structures by focused electron beam induced deposition holds great potential for the first-time realization of geometries that have so far been inaccessible by other means, at least at the resolution level which is achievable by this technique. In this way, fundamental studies relating to 3D artificial magnetic lattice structures are becoming feasible. However, the 3D nature of the constituent structural elements of, roughly, cylindrical shape will often lead to complex switching behavior associated with curling magnetization states. This has to be taken into account within simplifying model assumptions that rely on uniform macro-spin states. In addition, continuing efforts are required to reach the highest possible level of growth control in FEBID to be able to get the best possible agreement between the 3D target structure and actually grown nano-architecture. One may state that the results obtained so far in this endeavor are very promising indeed. 

## Abbreviations

3D (2D)	Three (Two)-Dimensional
FEBID	Focused Electron Beam Induced Deposition
SEM	Scanning Electron Microscopy
TEM	Transmission Electron Microscopy
EELS	Electron Energy Loss Spectroscopy
